# Chromoanagenesis: cataclysms behind complex chromosomal rearrangements

**DOI:** 10.1186/s13039-019-0415-7

**Published:** 2019-02-11

**Authors:** Franck Pellestor

**Affiliations:** 1 Unit of Chromosomal Genetics, Department of Medical Genetics, Arnaud de Villeneuve Hospital, Montpellier CHRU, 371, avenue du Doyen Gaston Giraud, 34295 Montpellier cedex 5, France; 2 INSERM 1183 Unit «Genome and Stem Cell Plasticity in Development and Aging », Institute of Regenerative Medicine and Biotherapies, St Eloi Hospital, Montpellier, France

**Keywords:** Chromoanagenesis, Chromothripsis, Chromoanasynthesis, Chromoplexy, Cancer, Evolution, Genome instability, Micronucleus, Double-strand breaks, Telomeres, Replication

## Abstract

**Background:**

During the last decade, genome sequencing projects in cancer genomes as well as in patients with congenital diseases and healthy individuals have led to the identification of new types of massive chromosomal rearrangements arising during single chaotic cellular events. These unanticipated catastrophic phenomenon are termed chromothripsis, chromoanasynthesis and chromoplexis., and are grouped under the name of “chromoanagenesis”.

**Results:**

For each process, several specific features have been described, allowing each phenomenon to be distinguished from each other and to understand its mechanism of formation and to better understand its aetiology. Thus, chromothripsis derives from chromosome shattering followed by the random restitching of chromosomal fragments with low copy-number change whereas chromoanasynthesis results from erroneous DNA replication of a chromosome through serial fork stalling and template switching with variable copy-number gains, and chromoplexy refers to the occurrence of multiple inter-and intra-chromosomal translocations and deletions with little or no copy-number alterations in prostate cancer. Cumulating data and experimental models have shown that chromothripsis and chromoanasynthesis may essentially result from lagging chromosome encapsulated in micronuclei or telomere attrition and end-to-end telomere fusion.

**Conclusion:**

The concept of chromanagenesis has provided new insight into the aetiology of complex structural rearrangements, the connection between defective cell cycle progression and genomic instability, and the complexity of cancer evolution. Increasing reported chromoanagenesis events suggest that these chaotic mechanisms are probably much more frequent than anticipated.

## Background

Over the past decade, genome sequencing effort combining new generation DNA sequencing technologies and efficient bioinformatics tools have lead to the discovery of new types of complex and massive chromosomal and genomic alterations characterized by the simultaneous occurrence of multiple structural rearrangements confined to one or a few chromosomal segments through a single catastrophic event. Grouped under the term of chromoanagenesis (for chromosome rebirth), this new class of genomic alterations involve 3 distinct phenomenons: the chromothripsis, the chromoanasynthesis and the chromoplexy [1].

The concept of chromoanagenesis provides new insight into the nature of complex chromosomal rearrangements. Both the complexity and the diversity of chromoanagenesis-related rearrangements raise important questions concerning the cellular mechanisms driving chromoanagenesis events, the aetiology of these chaotic processes and their impact in human pathology. Experimental models allowed to validate the existence of these catastrophic phenomenon, and to evidence some of the causative mechanisms. In this review, are summarized exciting data and recent progress on understanding the formation and the consequences of these complex genomic alterations.

## Chromothripsis

**Fig. 1:**
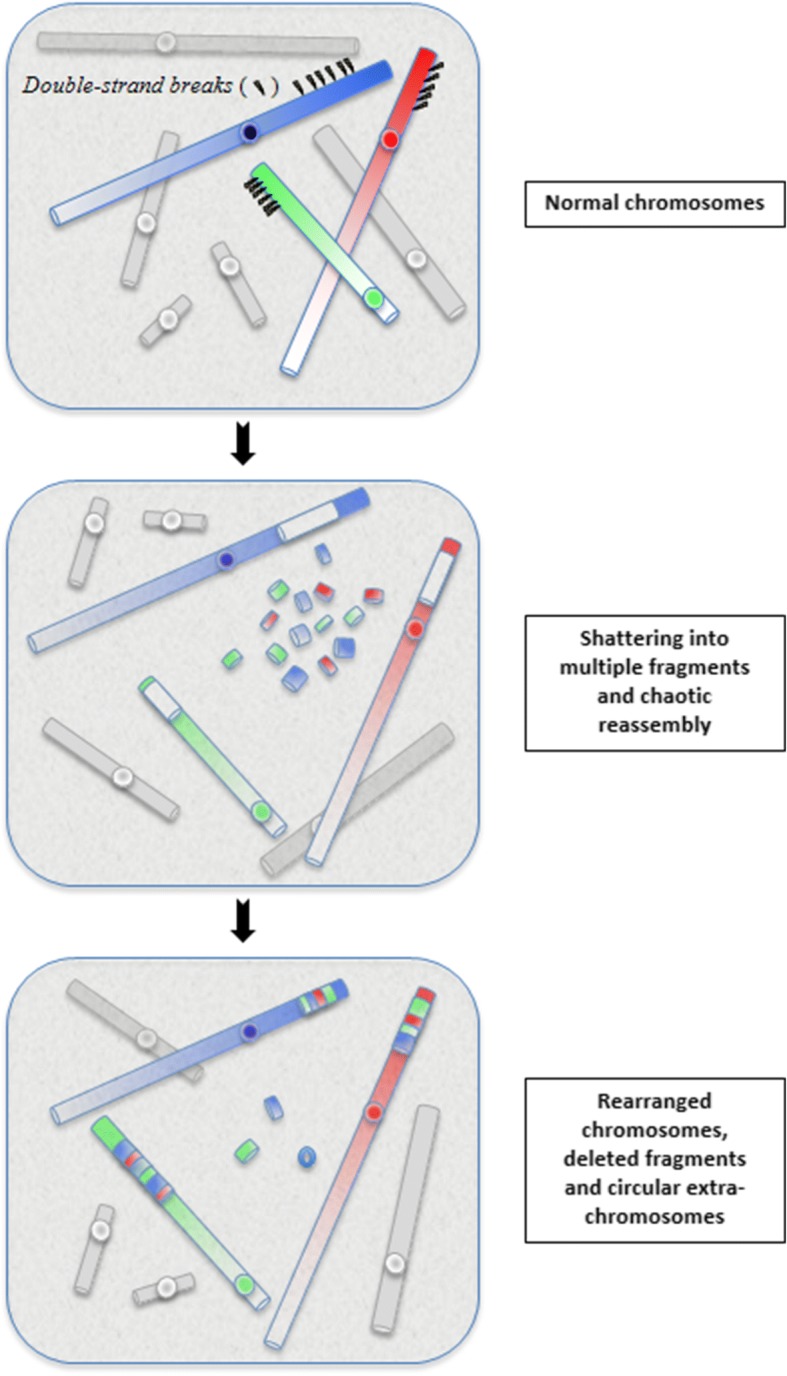
The concept of chromothripsis: during a one-step catastrophic event, multiple double-strand breaks occurred, restricted to a simple chromosomal segment or to a few closed chromosome domains, leading to the pulverization of chromosomal fragments. This shattering can produce tens to hundreds DNA fragments. Most of them are stitched back together by non-homologous end-joining (NHEJ), resulting in chaotic derivative chromosome(s), whereas some are lost or combined in small circular extra-chromosomes

Chromothripsis is the first of these new catastrophic process (mechanism) described in 2011 [2]. The phenomenon is currently defined as a mutational event driven by multiple double-strand breaks (DSBs) occurring in a single catastrophic event between a limited numbers of chromosomal segments, and followed by the reassembly of the DNA fragments in random order and orientation to form complex derivative chromosomes (Fig. 1).

Several factors common to all chromothripsis events, such as the generation of numerous clustered chromosomal breakpoints, the low DNA copy number changes and the preservation of heterozygosity in the rearranged segments, allow to distinguish chromothripsis from other complex chromosomal rearrangements and define its molecular signature [3, 4].

Initially described in cancers [1], the phenomenon was rapidly evidenced in patients with congenital abnormalities [5–7]. Notably, even some translocations and inversions classified as simple balanced rearrangements were identified as more complex than previously appreciated [8]. In the same way, extreme balanced germline chromothripsis were identified in patients with autism spectrum disorders and other developmental abnormalities [9, 10]. Also, chromothripsis was observed in healthy subjects [11, 12] as well as in prenatal diagnosis [13]. Some studies reported the possible reversibility of chromothripsis [14] and its potential curative effect [15]. Accumulating data on familial chromothripsis validated the notion of the heritability of some chromothripsis rearrangements.

Precise analysis of breakpoint junction sequences have indicated that the re-assembly of DNA fragments was driven by recombination-based mechanism such as classical non-homologous end joining (c-NHEJ) or alternative form of end joining (alt-EJ), operating in all phases of the cell cycles and working independently of micro-homologies but potentially error-prone [16–19]. Since the end-joining process mediates the formation of reciprocal translocations and complex three-way translocations, Kloosterman et al. [20] suggested that a similar cascade mechanism could operate in the creation of the derivative complex chromosomes found in constitutional chromothripsis.

Concerning the shattering of chromosome segments, multiple DBSs can arise from various exogenous sources such as ionizing radiation, free radicals, environmental toxins or chemotherapeutic drugs [21]. Even cannabis exposure has been associated with chromothripsis occurrence [22]. Other exogenous causal factors might be certain viral integration such as human papillomavirus (HPV) that can promote genomic instability and multiple DNA breaks [23]. Analysis of the aetiology of chromothripsis has also led to the identification of several cellular mechanisms capable of initiating chromothripsis process. Tubio and Estivill [24] proposed that chromothripsis might be caused by abortive apoptosis. Whereas apoptosis was considered as an irreversible cascade of extensive chromatin fragmentations leading to cell death, a small subset of cells could undergo a restricted form of apoptosis and thus survive. The partial DNA fragmentation could be restricted to regions of high chromatin accessibility. The subsequent DNA repair might be accomplished through a fast and incorrect repair process, promoting the emergence of chaotic chromosomal rearrangement [16, 25].

Since many examples of chromothripsis rearrangements affect chromosome ends, it has been proposed that chromothripsis could also arise via telomere attriction [2, 26]. Indeed, uncapped chromosome-ends are prone to fusion, leading to the formation of dicentric chromosomes [27]. During mitosis, this telomere crisis can yield complex rearrangements through breakage-fusion-bridge (BFB) cycles [28]. Several studies have suggested the association between chromothripsis and the occurrence of BFBs [26, 29]. By examining the fate of dicentric human chromosomes, Maciejowski et al. [30] evidenced the formation of chromatin bridges connecting daughter cells. These bridges can undergo nuclear envelope rupture and nucleolytic attack by cytoplasmic TREX1 exonuclease, causing in the restricted area of the bridge, chromothripsis-like rearrangements frequently associated with local hypermutations known as kataegis [30, 31].

Other proposed models suggest that replication stress and mitotic error could synergize to induce chromosomal instability and chromothripsis occurrence [16, 32, 33] or that premature chromosome condensation (PCC) induced by the fusion of an interphasic cell with a metaphasic cell could initiate chromothripsis, leading to incomplete replication and subsequent partial pulverization of chromosomes [34].

The emergence of chromothripsis has also been strongly associated with dysregulation or loss of p53 tumour suppressor genes. Known as the guardian of the genome, p53 plays a major role in maintaining genome stability by mediating cell cycle arrest, apoptosis and cell senescence in response to DNA damages [35, 36]. The potential implication of p53 pathways in chromothripsis occurrence was postulated by Rausch et al. [37] after the discovery of a striking correlation between germline p53 mutations (Li-Fraumeni syndrome) and chromothripsis patterns in patients with Sonic-Hedgehog medulloblastoma brain tumors. These findings led the authors to propose that germline p53 mutations could either predispose cell to catastrophic DNA rearrangements or facilitate cell survival after these catastrophic events.

An attractive mechanistic explanation to link all these causal processes with the confined nature of damages created during chromothripsis, is that the implicated chromosome(s) can be incorporated into a micronucleus in which chromothripsis-related damages will occur. Micronuclei are generally considered as passive indicators of chromosomal instability [38]. Crasta et al. [39] provided the first experimental evidence on this mechanism by the generation of micronuclei in several human cell lines and the subsequent observation of extensive genomic rearrangements during the cell cycles following the formation of micronuclei. Micronuclei display a double-membrane similar to regular nuclei, but micronuclei often undergo defective nuclear envelope assembly and the number of nuclear pore complexes (NPCs) is often inadequate. Recently, Liu et al. [40] showed that only “core” nuclear envelope proteins assemble efficiently around lagging chromosomes whereas “non-core” nuclear envelope proteins, especially NPCs, do not. This situation leads to a defect in the micronuclear import of essential components for DNA repair and replication, and consequently to reduced functioning in micronuclei. The chromatin sequestrated in micronuclei can undergo defective replication, resulting in the formation of complex rearranged chromosomes [41]. Micronuclei may persist in daughter cells over several cell cycles before being eliminated or reincorporated into the regular nucleus [42]. An additional pathway for the occurrence of DNA damages in micronuclei is the irreversible breakdowns of their membranes during interphase. Zhang et al. [43] proposed that membrane rupture enables enzymes such as endonucleases or topoisomerases to act aberrantly on micronuclear chromosome fragments. The entry of the cell into mitosis while the micronucleus is still undergoing DNA replication will result in micronuclear DNA pulverization due to premature chromosome compaction, and the subsequent chaotic reassembly of chromosome fragments [39, 44].

Using an elegant in vitro model to specifically induce mis-segregation of the Y chromosome, Ly et al. [45] observed frequent Y chromosome sequestration into micronuclei, followed by shattering and incorrect reassembly of Y chromosome fragments through 3 consecutive cell cycles. By using inhibitor of DNA repair, the authors demonstrated that NHEJ mechanism was not efficient in the micronucleus, but operated during the subsequent interphase, after the incorporation of Y chromosome fragments in a daughter nucleus.

These micronucleus-based models have the potential to explain many features of chromothripsis, especially how such massive damages can be confined to one or just a few chromosomal segments [46].

## Chromoanasynthesis

As investigations on the aetiology of chromothripsis events progressed, it became clear that the chromothripsis mechanism could not account for all the phenomenon of chaotic and rapid genomic rearrangements. Indeed, a number of complex rearrangements with duplication and triplication cannot be explained by NHEJ-mediated repair mechanisms. This led to the proposal that chaotic rearrangements could also result from another one-off cellular event in which there are copy number increases. This distinct process has been identified and named chromoanasynthesis, for chromosome reconstitution [47]. Although its molecular mechanism differs from that of chromothripsis, its biological consequences are similar, with the rapid formation of highly remodelled chromosomes. To date, most of patients with chromoanasynthesis-mediated rearrangements display developmental delay, intellectual disability and dysmorphic features, but individuals with relatively mild phenotypic effects have also been described [48, 49].

Chromoanasynthesis is considered to be a replication-based complex rearrangement process that involves serial fork stalling and template switching (FoSTeS) or microhomology-mediated break-induced replication (MMBIR) mechanisms [50, 51].

Numerous exogenous or endogenous agents can create conditions of replication stress by interfering with the progression and the stability of the replication fork [52, 53]. In a stressing situation, when the replication forks stall or pause in the vicinity of DNA lesions, fragile sites, cluster of tightly bound proteins or structural domains that are difficult to replicate, such replication stress may lead to aberrant replication with the use of alternate error-prone DNA repair mechanisms such as FoSTeS and MMBIR that lead to the formation of complex structural changes and copy number variations [54].

**Fig. 2:**
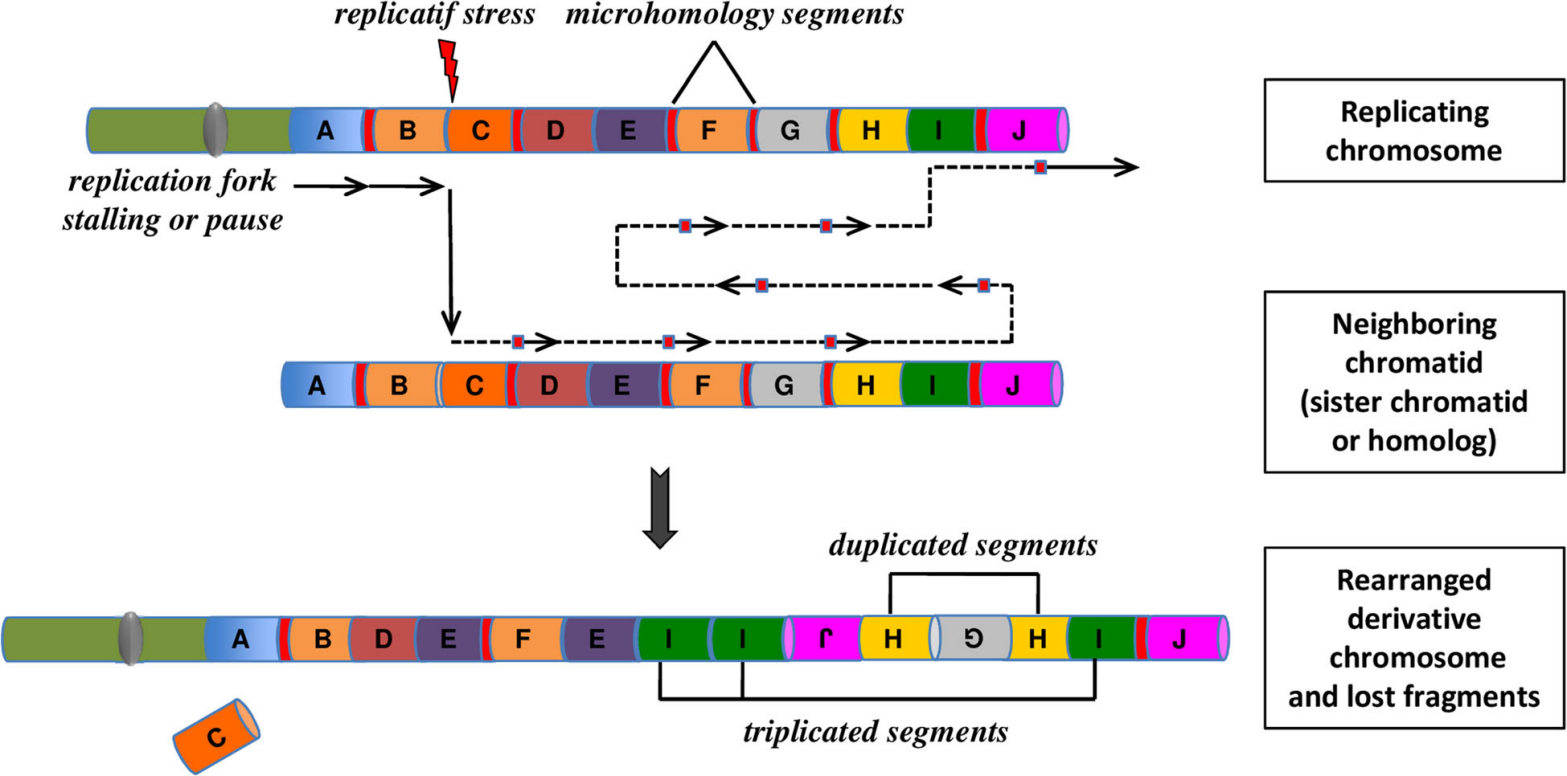
The concept of chromoanasynthesis: the phenomenon can arise when a replication fork stall or collapse. The lagging strand of the defective fork disengage and a series of micro-homology-dependent template and switching events occur with other replication forks in physical proximity, before the completion of DNA synthesis on the original template. The process leads to the formation of complex genomic rearrangements that typically involves duplications and triplications. The insertion of short nucleotide sequences (3–5 bp) at breakpoint junctions provides evidence for a replication-mediated process. Two mechanisms, Fork Stalling and Template Switching (FoSTeS) and Microhomology-Mediated Break-Induced Replication (MMBIR), have been identified as responsible for this process of massive genomic rearrangement

In the models of FoSTeS and MMBIR, the lagging DNA-strand end can serially disengage and switch to another nearby template. DNA would then be copied by another active replication fork. The new template strand is not necessarily adjacent to the initial replication fork, but in 3D physical proximity. Multiple fork disengaging, and strand invasions can occur before the resumption of replication on the original template (Fig. 2).

Like chromothripsis, chromoanasynthesis events involve a combination of structural rearrangements. However, the occurrence of localized multiple copy-number changes, particularly region-focused duplication and triplication and short stretches of micro-homologies at the breakpoint junctions, are both the hallmarks of replication-based mechanism with iterative template switches and define the chromoanasynthesis phenomenon. In addition, a high incidence of marker chromosomes has been reported in patients with chromanasynthesis disorders [55, 56].

Molecular situations responsible of replication fork stalling are numerous and a variety of cellular events may trigger the genome instability underlying chromanasynthesis events. All environmental insults and physiological pathway alterations that compromise genome stability, may potentially give rise to replication stress and subsequent chromoanasynthesis occurrence [57].

Such replication-based mechanisms do not necessarily require micronucleus formation to explain the occurrence of massive chromosomal rearrangements. However, the micronucleus-mediated models proposed for chromothripsis provide attractive cellular explanation also for chromoanasynthesis phenomenon.

## Chromoplexy

**Fig. 3:**
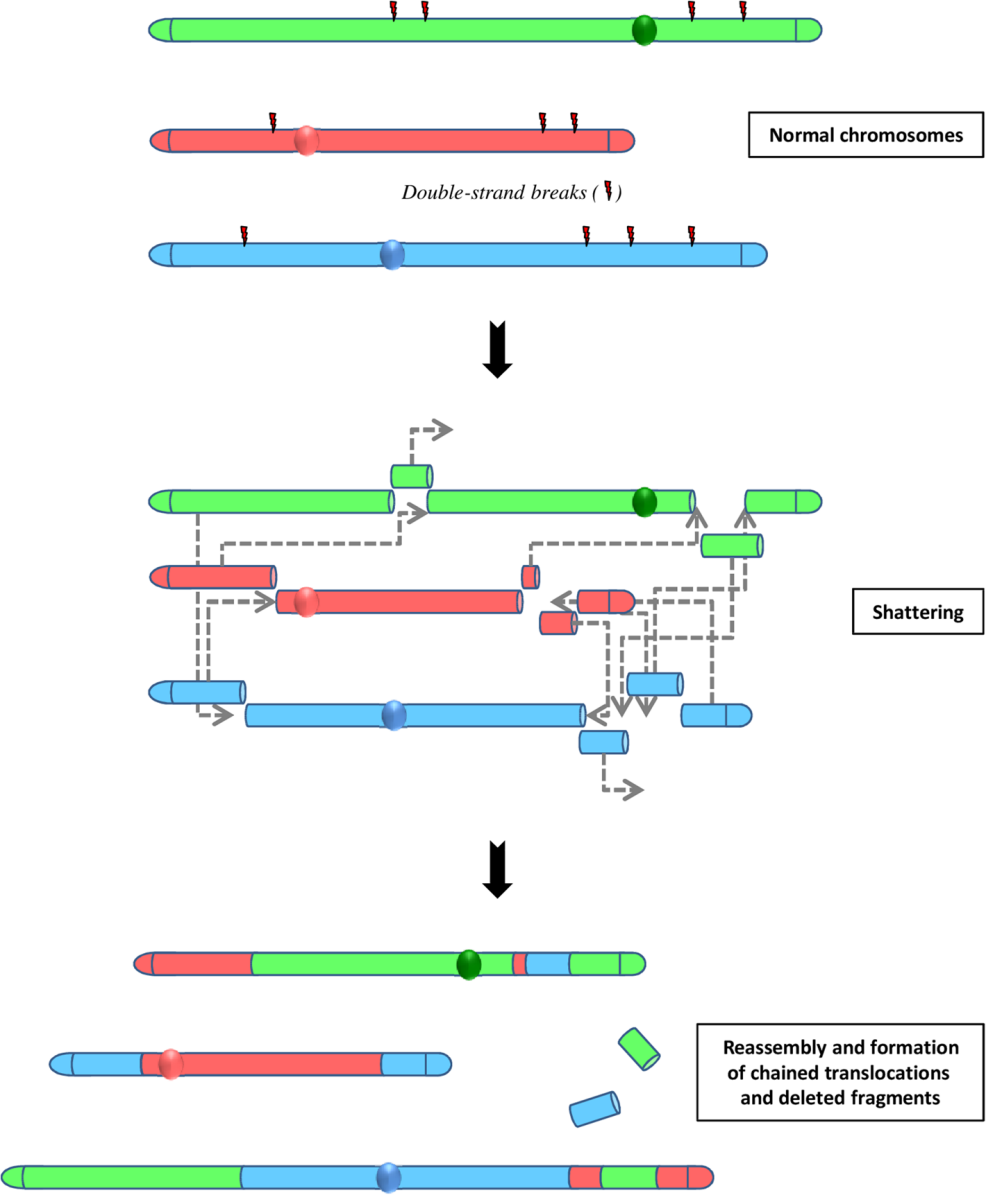
The concept of chromoplexy: a series of chained, complex inter- and intra-chromosome translocations, involving up to eight chromosomes with frequent deletions at their breakpoints and presumably occurring simultaneously. These serial rearrangements occur in the majority of prostate cancers. Non-homologous end-joining (NHEJ) is the predominant mechanism for reassembling then chromosomal fragments

A third type of massive rearrangement has been evidenced in prostate cancer. Termed chromoplexy [58], for chromosome restructuring, this phenomenon is characterized by the interdependent occurrence of multiple inter-and intra-chromosomal translocations and deletions (Fig.3).

The analysis of breakpoint sequences indicated that all the partner fragments involved in these serial rearrangements originate from DSB and exhibit deletion at the fusion junctions of chained rearrangements. Computational simulations revealed that chromoplexy events arise through a single catastrophic event.

These chains of rearrangements, numbering from 3 to over 40 and involving up to 7 chromosomes in a single chain, occur in a large majority of the prostate cancer studied. In contrast to chromothripsis, chromosomal rearrangements present little or no copy number alterations.

The involvement of the TMPRSS2-ERG gene fusion (EST+) in the complex event, suggest chromoplexy may arise from the same transcriptional-mechanism driven by the androgen receptor (AR) that induce TMPRSS2-ERG fusion. Thus, the nuclear co-localization of transcriptional sites could be mediated by the co-localization of androgen receptor [59]. AR-mediated transcription has been implicated in the occurrence of DSBs. In the context of chromoplexy-related process, the AR, by coordinating the induction of clustered DSBs, might effectively promote the formation of chained rearrangements within a restricted nuclear domain.

Sequence analyses of prostate tumour specimens revealed distinctive patterns of chromoplexy. Tumour harbouring oncogenic ETS fusion (ETS+) produced more inter-chromosomal rearrangements whereas tumour with a deletion of CHD1, a putative tumour suppressor gene, showed an excess of intra-chromosomal chained rearrangements. Chromoplexy could simultaneously dysregulate multiple cancer genes across the genome and contribute to the aggressive evolution of high-grade prostate cancers. The detection of similar chained rearrangements in cases of lung cancers, melanomas or neck cancers suggests that chromoplexy could occur in a larger spectrum of cancers [60].

Interestingly, these data concerning the chromoplexy process also supports the punctuated equilibrium model of cancer evolution [61].

## Factors operating in chromoanagenesis

What drives the cell in the choice of a DNA repair process? The question is particularly relevant since repair outcomes don’t always seem to be in line with the ultimate goal of preserving genome integrity. Firstly, this choice must be dictated by the cell-cycle position. NHEJ can occur at any stage of the cell cycle. In contrast, replicative repair pathways operate only during S-phase and eventually in G2. Of particular importance are the number of DSBs and the energy required by the cell to manage repairs on a short timescale. High loads of DSBs can rapidly saturate the repair machinery. Gudjonsson et al. [62] demonstrated that more than 20 DSBs can alter classical error-free repair pathways such as homologous recombination (HR), thus leading the cell to engage in faster but error-prone repair processes such as c-NHEJ or alt-EJ. It is also considered that the complexity of DSBs and the accompanying chemical alterations are determinants for the cellular choice of DSB repair pathway [63]. Specific sequence features may stimulate or simply predispose to the occurrence of complex genomic reassociations. Analyses at the junction points have evidenced the recurrent presence of unusual DNA sequences such as low-copy repeats (LCRs) or tandem repeats such as Alu or LINE sequences [5, 64]. In some chromothripsis and chromoanasynthesis breakpoints, the frequency of LCRs or LINEs is significantly higher than the genome-wide average [65]. These repetitive sequences constitute points of genomic instability and may serve as substrates for chromosomal rearrangements [66, 67]. For instance, the relatively long micro-homology (20–40 bp) shared by Alu sequences may facilitate template switching in replicative repair processes [68]. Other particular elements, such as palindromic sequences, have the potential to form distinct secondary structures, which are able to impede replication and stimulate DSBs [69]. In chromoanasynthesis investigation, Liu et al. [47] demonstrated that complex genome architecture such as hairpin structure, palindromic sequences or low-copy repeat clusters may confuse the DNA replication machinery and exacerbate serial template switching events driven by FoSTeS and MMBIR.

The chromatin structure also regulates DSB repair through histone modifications and nucleosome remodeling within approximately 50 kb on each side of DSBs, in order to promote the formation of an open, relaxed chromatin structure at the DSBs and to facilitate loading of DNA repair proteins [70, 71]. With regards to this process, the compact heterochromatin is less accessible and potentially less sensitive than euchromatin to DNA damaging agents that initiate DSBs. A plausible connection between chromothripsis and fragile sites has also been postulated [72]. Fragile sites contribute to genomic instability [73] and thus they can be preferential sites for the multiple breakage events observed in chromothripsis and chromoanasynthesis events.

Recent reports provided evidence that transposable elements can serve as drivers in the formation of chromoanagenesis by compromising the genomic stability and facilitating chromatin conformation changes and DNA breaks [74, 75]. The presence of transposable elements within the genome is currently associated with chromosome restructuring [76], genetic disorders [77] and chromosome evolution [78]. The abundance of these elements in the human genome provides numerous potential substrates for microhomology-mediated template switching and chromoanasynthesis occurrence.

Lastly, one could speculate than the genomic architectural feature is a determining factor in chromoanagenesis emergence. The detection of chromothripsis on unique chromosomal regions has suggested that shattering and reassociation might occur when chromosomes are highly condensed, i.e. during mitosis. However, examples of constitutional chromoanagenesis-compatible events implicating several chromosomes indicate that the phenomenon may preferentially arise during interphase when chromosomes are relaxed throughout the nucleus.

The general consensus is that interphase chromosomes are compartmentalized in discrete but dynamic nuclear territories that could confine intranuclear processes to a limited space [79, 80]. This view could imply the spatial proximity of chromosomes involved in chromothripsis and their proximity with potential discrete domains that cluster DNA repair factors [81, 82].

Accumulating date from chromosome configuration capture (3C)-based approaches and the analysis of topologically associated domains (TADs) provide information on cell-to-cell architecture variability and indicate how structural rearrangements in the layout of genes and their regulatory sequences can lead to ectopic gene interactions and altered gene expression [83–85]. HI-C experiments and TADs analysis performed on induced pluripotent stem cells (iPSCs) derived from a patient with a de novo germline chromothripsis have allowed to evidence how the chromothripsis rearrangements led to gene dysregulation and contribute to the patient’s complex congenital phenotype [86]. The occurrence of massive chromosome rearrangements can lead to deleterious genomic configurations but also to genetic novelty, with the formation of stable and inheritable rearranged genomic constitution. In addition to 3D genome organization, another important factor could be the movement of chromatin within the nucleus. Certain genes seem to have preferential contacts with their neighbours in a phenomenon termed “chromosome kissing” [87]. Local movements of DSBs have been evidenced in yeast [88] and *Drosophilia* [89] where DSBs within heterochromatic domains have to move to a more euchromatic environment to be repaired. Certain experiments in mammalian cells have suggested limited mobility for DSBs [90], whereas others have shown the intranuclear repositioning of derivative chromosomes and their normal counterparts in translocation cell lines [91]. To reconcile this large range of data, Dion and Gasser [92] proposed that different types of DNA damages lead to different modes of nuclear movements, depending on how the lesion is repaired. The localization of chromothripsis restricted to a single chromosome segment or to a small nuclear territory could establish the upper limit of tolerance of the phenomenon by the cell. Further works are needed to discover if chromatin mobility and its proximity with some elements of nuclear architecture (such as nucleolus, nuclear matrix, lamina) could be a limitative factor in case of accumulation of DSBs.

Whatever parameters are implicated in the emergence of chromoanagenesis events, the high probability of error in repair processing as well as in replication mechanisms suggests that cells have developed tolerance for sequence modifications at the breakpoint junctions. The logic underlying the emergency of chromoanagenesis events could be more the preservation of genomic stability than genomic integrity.

## Chromoanagenesis in cancer

Chromothripsis was originally described in a case of chronic lymphocytic leukemia in a female patient displaying 42 somatic acquired structural rearrangements on the long arm of chromosome 4 [2] The discovery of such a pattern of massive interchromosomal rearrangements was made by combining next-generation paired-end sequencing and single nucleotide polymorphism (SNP) assays. To date, chromothripsis has now been described in a broad spectrum of human cancers including neuroblastoma, medulloblastoma, myeloma, retinoblastoma, colorectal cancers, or hematologic malignancies [93–99]. A survey of 4934 cancers indicated that chromothripsis was found in 5% of all samples, with incidences ranging from 0% in head carcinoma to 16% in glioblastoma [100]. A large analysis of 8227 cancers revealed the occurrence of chromothripsis-like massive rearrangements in 1 to 2% of the sample [101]. Through a large-scale analysis of more 22.000 tumoral array data sets covering 132 cancer types, Cai et al. [102] evidenced the heterogeneity of the genome aberrations patterns associated with chromothripsis-like events. Altogether, these data provide evidence that at least 2 to 4% of all human cancers involve chromothripsis events, affecting one or several chromosomes. Glioblastoma and bone tumours appear to be the most affected types of cancer with up to 39 and 25% of chromothripsis [103].

In all cases, chromothripsis is associated with aggressive forms of cancer and poor patient survival [104, 105]. According to the type of cancer, specific chromosomes have been identified to be more sensitive to chromoanagenesis events [98, 102]. Thus, chromothripsis was more frequently detected in genomic regions containing critical gene for the DNA repair, the cell cycle regulation or the proliferation [106]. In some tumoral chromothripsis, the chaotic reorganization may lead to the generation of circular, extra double-minute chromosome markers that often include oncogenes and are frequently amplified [37, 107]. This may contribute to substantial changes in copy number state as well as the chromosomal instability in tumoral cells [1]. Another example of the contribution of chromanagenesis to tumoral evolution is the formation of neochromosomes, giant extra-chromosomes found in 3% of cancers, which associates chromoanagenesis events and BFB cycles [108, 109]. However, chromothripsis was also observed in uterine leiomyomas, a common and low-malignant smooth-muscle tumour, indicating that chromothripsis does not systematically have a dramatic oncogenic effect [110].

TP 53 mutations have been associated with chromothripsis in medulloblastoma and leukemia [37, 111]. High prevalence of chromothripsis events was also reported in patients with Ataxia Talangiectasia [99], indicating that alteration affecting other essential pathway for the maintenance of genome stability and cell cycle progression, such as ATM function, can also trigger chromothripsis occurrence. Also, studies of retinoblastoma progression indicated that chromothripsis can initiate tumorogenesis by inactivating a tumour suppressor gene [97]. Complex breakpoints in cancers may also exhibit significant array of short sequences derived from distinct loci, suggesting replication-based mechanisms consistent with chromanasynthesis events [112]. Collectively, these data suggest that cancer-associated chromoanagenesis rearrangements are more complex and subtle than previously envisaged, with the creation of various oncogene lesions, loss or disruption of tumour suppression genes and the construction of oncogenic fusions. Alterations in oncogenes or tumour suppressors that destabilize the genome can induce chromosome lagging and micronuclei formation. The formation of micronuclei containing whole chromosome(s) or chromosome fragments has been documented for many years as a frequent hallmark of genome instability in cultured tumoral cells [113], but we do have precise information on their real in vivo frequency.

The long-standing paradigm that genome alterations in cancer arise through the progressive accumulation of mutation has been deeply challenged by the discovery of chromoanagenesis events that might constitute major mutational gamers. Thus, in pancreatic cancer, the observation that two-thirds of tumours harboured complex chromothripsis-like patterns has contributed to the notion that pancreatic cancer progression was not gradual [98]. The concept that cancer genome can evolve in rapid bursts is consistent with the evolutionary model of punctuated equilibrium (see chapter below).

## Chromanagenesis and evolution

Beyond the impact of chromoanagenesis events as pathogenic mechanisms, an interesting question is the potential driving role of these phenomenon in species evolution. The occurrence of chromoanagenesis event appears to be in good agreement with macroevolution models such as the “hopeful monster” model [114] or the “punctuated equilibrium” theory [115] proposed as a complement to phyletic gradualism. These models postulated that species undergo little variations during most of their evolutionary history, interrupted by rare but abrupt bursts of change leading to the subsequent emergence of new species. During the last decade, accumulated data have demonstrated how genetic and chromosomal alterations can have huge impacts in developmental evolution. Numbers of studies have documented punctuated equilibrium and hopeful monsters in various species, introducing the notion of “transgressive segregations” for the generation and the rapid fixation of new genotypes in population. Prominent models argue that chromosomal rearrangements reduce gene flow through their suppressive effect on recombination [116]. Complex rearrangements such as chromoanagenesis events may modify gene position relative to replication origins or lead to the generation of new linkage gene block or new chimeric genes. Several models of chromosomal speciation are thus based on the existence of gametic barriers resulting from the fixation of various genomic rearrangements in a population [117]. Thus, in the gibbon genome, the insertion of a retro-transposon named LAVA in genes implicated in cell cycle progression and chromosome segregation appears to be at the origin of a high rate of chromothripsis-like rearrangements leading to the accelerated evolution of the gibbon karyotype and the emergence of different gibbon lineages [118, 119]. Another example of genome reshuffling and speciation driven by massive chromosome rearrangements is the extensive chromosome reshuffling observed in the marsupial family *Macropodidae*, with numerous interchromosomal rearrangements [120]. In 2007, Crombac and Hogeweg [121] demonstrated that genome restructuring mediated by massive chromosomal rearrangements was a beneficial operator for shorty-term adaptations to a new environment. Chromoanagenesis events as processes for rapid and profound genomic modification could be regarded as credible molecular mechanisms for evolutionary changes.

## Conclusion

Undoubtedly, chromothripsis, chromanasynthesis and chromoplexy are among the most unexpected biological discoveries made from the high-resolution genome analysis. The identification of these 3 unanticipated catastrophic phenomenon has deeply modified our perception of the genesis and the aetiology of complex genomic rearrangements. The investigation of this new class of genomic alterations has also provided new and important insights on the mechanisms connecting defective cell cycle progression with cellular stress and genomic instability, the role of genome maintenance pathways and the capacity of cells to manage such crisis phenomenon [122, 123]. This found expression in the causal link between disordered mitotic progression and the occurrence of complex structural rearrangements via the micronuclei-based process.

All these data support the idea that the occurrence of chromoanagenesis events in the genome is grossly underestimated and that extremely complex but balanced structural rearrangements can be tolerated in human germline and compatible with viability [124]. To date, the existence of chaotic genomic alterations is not restricted to human but there are also documented in other mammalians [118, 120] in plants [125], in nematode *Caenorhabditis elegans* [126], and *Saccharomyces cerevisiae* [127], emphasizing the notion that the cellular pathways responsible for generating such highly complex patterns of chromosomal rearrangements are highly conserved.

The identification of chromoanagenesis phenomenon in both cancers and congenital disorders provides a new perception of how genomes can be rapidly altered. Despite a high incidence of cell death during the process, the formation of chaotic genomes could represent a powerful survival strategy for the genome when under crisis, and chromoanagenesis-mediated events could constitute inherent mechanisms for maintaining genome stability and integrity [128, 129].

## Abbreviations

BFB: Breakage-fusion-bridge
DSB: Double-strand break
FoSTeS: Fork stalling and template switching
HPV: Human papillomavirus
HR: Homologous recombination
iPSC: Induced pluripotent stem cell
LCR: Low-copy repeat
MMBIR: Microhomology-mediated break-induced replication
NHEJ: Non-homologous end joining
NPC: Nuclear pore complex
PCC: Premature chromosome condensation
TAD: Topologically associated domain
